# Intraoperative Cisternostomy during Cerebellopontine Angle Tumor Surgery: A single centre experience without Postoperative Cerebrospinal Fluid Diversion

**DOI:** 10.12669/pjms.41.13(PINS-NNOS).13377

**Published:** 2025-12

**Authors:** Tariq Imran Khokhar, Abdul Ghafoor, Haseeb Mehmood Qadri, Hamza Toheed, Muhammad Nauman Hasan

**Affiliations:** 1 Tariq Imran Khokhar, MBBS, FCPS. Punjab Institute of Neurosciences, Lahore, Pakistan; 2 Abdul Ghafoor, MBBS, FCPS. Punjab Institute of Neurosciences, Lahore, Pakistan; 3 Haseeb Mehmood Qadri, MBBS. Punjab Institute of Neurosciences, Lahore, Pakistan; 4 Hamza Toheed, MBBS. Punjab Institute of Neurosciences, Lahore, Pakistan; 5 Muhammad Nauman Hasan, MBBS, MS. Punjab Institute of Neurosciences, Lahore, Pakistan

**Keywords:** Acoustic neuroma, Brain tumors, Cerebellopontine angle, Cisternostomy, Hydrocephalus, Pakistan

## Abstract

**Background and Objective:**

Cerebellopontine angle (CPA) is an important anatomical landmark hosting important neurovascular structures. Hydrocephalus presents a great challenge in CPA lesions management. We aimed to highlight the significance of intraoperative cisternostomy and without the need of preoperative and postoperative cerebrospinal fluid (CSF) diversion.

**Methodology:**

This retrospective observational study was conducted at Punjab Institute of Neurosciences involving consecutive, single-surgeon led cases of CPA space occupying lesions (SOL) between 2022 and 2024. Data was collected for demographic and clinical presentation, extent of resection and the need for post-operative CSF diversion while making use of intraoperative cisternostomy in all cases.

**Results:**

A male preponderance of 51.90% (42) patients was identified with an overall mean age of 39.13±13.18 years. Eighty-one patients underwent intra-operative cisternostomy of which 98.80% (80) cases were adult patients. The commonest clinical manifestations at presentation were headache in 91.4% (74) and sensorineural hearing loss in 92.60% (75) cases. Mean preoperative Glasgow coma scale (GCS) score was 14.9±0.6. Mean pre-operative KPS (Karnofsky performance status) score was 81.35 ± 13.1. About 91.40% (74) patients underwent gross total resection. Schwannoma was seen in 80.2% (65) patients making it the most common CPA pathology. Mean immediate postoperative GCS was 13.93±2.26. Mean postoperative KPS score was 78.76±15.11. Only 13.80% (12) patients required post-operative CSF diversion in the form of ventriculoperitoneal (VP) shunt during first year of follow up.

**Conclusion:**

Retrosigmoid approach with intraoperative cisternostomy for excision of cerebellopontine angle lesions is a safe and efficient approach with better outcomes, mitigating the need for pre-operative and post-operative CSF diversion.

## INTRODUCTION

Cerebellopontine angle (CPA) is an important anatomical landmark which harbors cerebellopontine angle cistern which contain important neurological and vascular structures.^1^ Tumors in the CPA are mostly benign consisting of 5-10% of all intracranial tumors, most common of which are schawannomas (75-85%), meningiomas (10-15%) and epidermoid cysts (8%).^1^,^2^ The clinical manifestations of patients with CPA space occupying lesion (SOL) are of hearing loss, tinnitus, dizziness and signs of hearing loss or basal cranial nerve deficits. Treatment options for CPA lesions include observation, radiotherapy or microsurgery.

Complications related to surgery include hydrocephalus, CSF (cerebrospinal fluid) leak, cranial nerve palsy, hemorrhage. Hydrocephalus presents a great challenge in CPA lesions management. It greatly effects preoperative, intraoperative and postoperative management and patient recovery. It occurs in 3.7-42% patients of vestibular schwannomas.^3^ Management of hydrocephalus greatly impacts on patient outcome. There are multiple methods to address hydrocephalus in patients with cerebellopontine angle lesions. Keulen et al. recommend preoperative CSF diversion in patients with hydrocephalus followed by definitive surgery.^4^ Some authors suggest postoperative ventriculoperitoneal (VP) shunting. Other methods include third ventriculostomy, external ventricular drainage.^5^-^7^ Another method that can be used for managing hydrocephalus in these patients is intraoperative cisternostomy. We believe that this approach can be very helpful for surgeon intraoperatively and can save patient subsequent surgery requiring CSF diversion. We will study patients undergoing excision of CPA SOL excision via retrosigmoid approach and intraoperative cisternostomy.

Utilizing this new approach for the resection of CPA SOLs could be a valuable addition to the surgical toolkit. A thorough search using PubMed Central and Google Scholar indicates a lack of studies globally—and particularly from Pakistan—that assess the advantages and limitations of the intraoperative cisternostomy without postoperative CSF diversion for resecting CPA tumors. This study aimed to highlight the clinicopathological characteristics and surgical outcomes of patients with CPA SOLs who underwent resection with intraoperative cisternostomy and without postoperative CSF diversion.

## METHODOLOGY

This is retrospective observational study, conducted in Punjab Institute of Neurosciences (PINS) Lahore including patients who underwent CPA SOL excision from 1^st^ January 2022 to 31^st^ December 2024. Retrosigmoid approach along with inferior cerebellopontine angle cisternostomy was used for excision of CPA tumors. Non-probability convenience type sampling technique was utilized. Data was retrieved from patient files, records and Picture archiving and communicating system (PACS). Data was collected via Google forms.

### Ethical approval:

It was granted by the Institutional Review Board, Punjab Institute of Neurosciences (IRB-PINS), with approval number 2035/IRB/PINS/Approval/2025, dated January 23, 2025.

### Inclusion criteria:

- Patients with age 14 years or above.
- Patients who underwent retrosigmoid suboccipital craniectomy with intraoperative cisternostomy or CSF diversion.
- Patients with at least three months of follow-up.

### Exclusion criteria:

- Patients who lost to follow up.
- Patients with incomplete records.

### Data analysis technique:

Data was retrieved from patient files, records and Picture archiving and communicating system(PACS). Data was collected via Google forms. Study focused on patient’s pre-operative and post-operative complaints, Glasgow coma scale (GCS), Karnofsky performance status (KPS) score, CSF diversion, radiological findings and histopathological diagnosis. Other variables included were age, gender and side predilection. Histopathology reports were collected. Data was analyzed using descriptive analysis including percentages, frequencies, measures of central tendencies (mean, mode, median) and central dispersion (standard deviation).

## RESULTS

Total 81 patients were included in this study. Mean age of participants was 39.13 ± 13.18 years. Male predominance of 51.9% (42) was seen, while 48.1% (39) were females. The commonest signs and symptoms were headache in 91.4% (74), focal cranial nerve deficit (hearing loss) in 86.4% (70) and vomiting in 18.5 % (15) patients (Table-I).

**Table-I:** Symptoms and signs at presentation of patients where total number of patients is N=81 expressed as percentages n/N.

Symptoms and signs at presentation	Percentage n/N (frequency, n)
Headache	91.4 (74)
Focal cranial nerve deficit	92.6 (75)
	Hearing loss 86.4(70)
	Facial weakness 22.2(18)
Vomiting	18.5(15)
Positive Cerebellar signs	3.6 (3)
Vertigo	2.5 (2)
Seizures	2.5(2)
Motor deficit	2.5 (2)
Loss of consciousness	2.5 (2)
Difficulty walking	1.2 (1)
Difficulty swallowing	1.2 (1)
Visual deterioration	1.2 (1)

Pre-operative mean KPS was 81.35 ± 13.1 while mean GCS was 14.9 ± 0.6 among which 98.7 % (81) had GCS score of 13-15. In 19.8% (16) patients pre-operatively CSF diversion was already performed and they were referred to our institute from other centers of Pakistan. Post operatively CSF diversion was done via VP shunting in 17.4 % (14) patients and extra-ventricular drainage (EVD) in 2.4 % (2). There was a slight right sided predominance 53.1 % (43). Amongst rest 45.7 % (37) were left sided and 2.5% (2) were bilateral. On magnetic resonance imaging (MRI), 67.9 % (55) were hypointense on T1 weighted images (T1WI) while 93.6 % (26) were hyperintense on T2 weighted images (T2WI) (Table-II).

**Table-II:** Magnetic resonance imaging (MRI) findings in patients where total number of patients is N=81 expressed as percentages n/N.

MRI Findings		Percentage n/N (frequency, n)
T1	Hypointense	67.9 % (55)
	Isointense	32.1 % (26)
	Hyperintense	0 (0)
T2	Hypointense	0 (0)
	Isointense	7.4 % (6)
	Hyperintense	92.6 % (75)
T1 with contrast	Heterogeneous	80.2 % (65)
	Homogeneous	16 % (13)
	Non-enhancing	3.7 % (3)

Retrosigmoid sub-occipital approach was opted for all the patients. Gross total resection (GTR) was achieved in 91.4% (74) patients, subtotal in 7.4 % (6) and near total in 1.2% (1). On histopathology, most of the lesions were schwannoma in 80.2 % (65), meningioma in 16 % (13), epidermoid cyst in 2.5 % (2) and pilocytic astrocytoma 1.2 % (1) cases. This highlights that the commonest neoplastic lesions arising in CPA are benign. Mean post-operative GCS was 13.93 ± 2.26 and KPS was 78.76 ± 15.11. In 25 (30.9%) patients, the presenting complaints were resolved and only 11 (13.8%) needed post-operative CSF diversion. Post-operatively gag reflex was weak in 33.1 % (27), facial weakness was observed in 11.1. % (9) and signs of raised intracranial pressure (ICP) were seen in 7.4 % (6) cases (Table-III).

**Table-III:** Postoperative signs and symptoms of patients where total number of patients is N=81 expressed as percentages n/N.

Post-operative signs and symptoms	Percentage n/N (frequency, n)
Absent gag	33.1 (27)
Facial weakness	11.1 (9)
Signs of raise ICP	8.6 (7)
Loss of consciousness	1.2 (1)
Tracheostomy	1.2 (1)
CSF leak	0 (0)

## DISCUSSION

### Anatomy and common lesions:

CPA represents a space bounded by the pons, anterior cerebellum, and petrous temporal bone.^8^,^9^ It is an important landmark anatomically and clinically as it is occupied by the CPA cistern. Furthermore, CPA houses a variety of lesions. Tumors in the CPA are mostly benign consisting of 5-10% of all intracranial tumors. Gender distribution is almost equal in CPA lesions. Most common histopathological type is schwannomas (75-85%), meningiomas (10-15%) and epidermoid cysts (8%).^1^,^2^,^10^ This is consistent with our study as schwannoma was most common histopathological lesion in 80.2% (65) cases followed by meningioma 16.0% (13) cases.

### Presenting signs and symptoms:

Patients with CPA tumors present with a myriad of non-specific symptoms. The most common include headache, sensorineural hearing loss, tinnitus, and dizziness and hydrocephalus in case of large lesions.^11^ These findings are consistent with our study as most common signs and symptoms were headache in 91.4% (74), hearing loss in 86.4% (70) and vomiting in 18.5% (15) cases.

### Investigations:

The diagnosis of CPA tumors is made based on history, physical examination, audiometric and radiological evaluation. Physical examination involves assessment of trigeminal, facial and vagus nerve. Pure tone audiometry (PTA) is used for assessment of hearing status. Radiological investigations include MRI and computerized tomography (CT). MRI is the gold standard for the diagnosis of CPA tumors. High-resolution computed tomography (CT) is useful to assess bony involvement. CPA lesions can be hypo intense, isointense and hyper intense on T1WI and T2WI weighted images of MRI. Vestibular schwannomas appear isodense on CT, hypo intense on T1WI and hyper intense on T2WI.^12^ Meningiomas appear hyper dense on CT, hypo intense on T1WI and hyper intense on T2WI.^12^ Differentiating features of meningioma from vestibular schwannoma include a hyper dense appearance on non-contrast CT, lack of internal auditory canal erosion, broad dural attachment, cleft of CSF between the tumor and brain parenchyma and thickening of dura around the tumor (dural tail sign).^2^ Our study also shows a similar trend on radiology.

### Surgical approaches:

**Fig.1:**
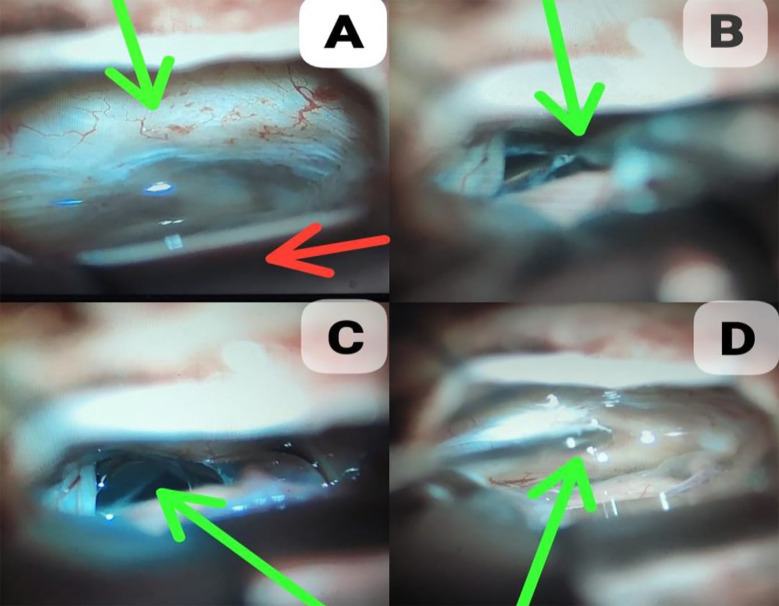
Intraoperative cisternostomy being performed under microscope for cerebellopontine angle space occupying lesion. A: Image shows the tentorium cerebelli (green arrow) and brain retractor (red arrow) with arachnoid mater visible in between appearing as a transparent membrane. B: Arachnoid knife (green arrow) used to dissect arachnoid mater. C: Arachnoid dissection complete. D: Sucker used to drain inferior cerebellopontine angle cistern.

**Fig.2:**
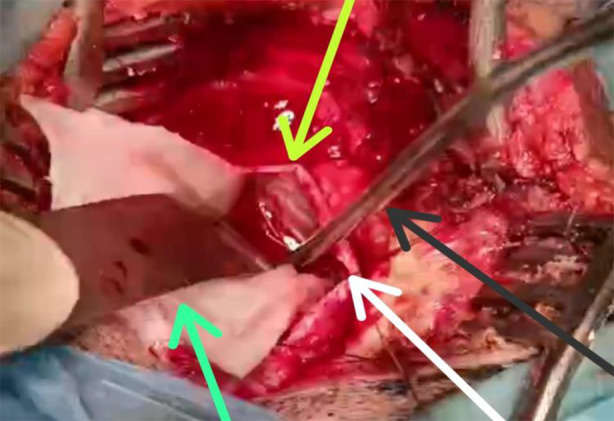
Naked-eye view of intraoperative cisternostomy to drain inferior cerebellopontine angle cistern (area pointed out with white arrow) using brain retractor (green arrow) and sucker (black arrow). Tied dura mater can be seen (yellow arrow).

There are multiple approaches to resect cerebellopontine angle lesions. Each approach has its own pros and cons. The choice of surgical procedure is based on the size of the tumor, the extension of the tumor within the canal, the surgeon’s preference, and baseline hearing function.^11^ Approaches utilized include retrosigmoid, translybranthine and middle fossa approach.^13^ Some authors suggest preoperative CSF diversion in patients with cerebellopontine angle lesions like external ventricular drainage (ETV) or supratentorial shunting.^5^,^14^ We have used retrosigmoid approach in all subjects. GTR was done in 91.4% (74) patients. Intraoperative cisternostomy was done in all patients. Inferior CPA cistern was targeted in all patients after opening of dura and retraction of cerebellum. The dura is first opened sharply at the inferior edge, placed just medial to the edge of the sigmoid sinus and just superior to the inferior bony margin. Inferior edge of dura is raised and pattie is passed over to protect cerebellum from retraction. Brain retractor is placed and the inferior CPA cistern is accessed via sharply opening the arachnoid (Fig.1 & Fig.2). This is most safely performed by opening the arachnoid just posterior to the ascending CN XI. CSF is drained until posterior fossa becomes relaxed and pulsatile.^15^

### Complications:

Complications related to surgery include hydrocephalus, CSF leak, cranial nerve palsy, hemorrhage. In our study, 33.1% (27) patients had absent or weak gag reflex postoperatively. Facial nerve palsy was present in 11.1(9) patients postoperatively highlighting the importance of this site, housing significant neurological apparatus. Hydrocephalus presents a great challenge in managing CPA lesions. Mechanism of development of obstructive hydrocephalus is well understood due to compression on fourth ventricle by tumor causing obstruction to CSF flow.^16^-^18^ It greatly effects preoperative, intraoperative and postoperative management and patient recovery. In a study, about 3.7-42% patients of vestibular schwannomas developed post-operative hydrocephalus.^3^

### Management of hydrocephalus:

Management of hydrocephalus greatly impacts patient outcome. There are multiple methods to tackle hydrocephalus in patients with cerebellopontine angle lesions. Keulen et al recommend preoperative CSF diversion in patients with hydrocephalus followed by definitive surgery.^4^ Some authors suggest postoperative VP shunting. Other methods include third ventriculostomy, external ventricular drainage.^5^-^7^ Our study focused on dealing with hydrocephalus in patients with CPA lesions. Another method that can be used for managing hydrocephalus in these patients is intraoperative cisternostomy. Anatomically cisterns that are easy to approach in retrosigmoid craniotomy are CPA cisterns and cisterna magna. We used intraoperative cisternostomy in all of our patients. Our results indicate that intraoperative cisternostomy helps in brain relaxation, preventing cerebellar contusions intraoperatively, aids in GTR of tumors and saves patient from subsequent CSF diversion surgery. Total of only 16 patients from our study population required postoperative CSF diversion in which 17.4%(14) had VP shunting and 2.4%(2) had EVD. A study carried out in Toronto also suggests that CPA tumor excision in patients with HCP can be done without need of postoperative CSF diversion where 13.9% of their study population had HCP and 78% of them didn’t require postoperative CSF diversion after tumor excision.^19^ In contrast our study emphasizes on intraoperative techniques like intraoperative cisternostomy for which current literature lacks studies both regionally and globally.

### Limitations:

Small sample size and retrospective data collection from patient’s record having missing information like tumor size, dimensions or volumetrics and has a tendency to introduce information and selection biases. Furthermore, due to a small sample size, the results of our study cannot be generalized to a larger population. The patients involved in the study are a single surgeon experience that may also serve as a limitation to our study. Prospective studies in this regard may help fellow clinicians in better management of CPA lesions. Our institution has already begun prospective study in this regard.

## CONCLUSION

Preoperative CSF diversion in CPA lesions can only be used in patients who present with emergent hydrocephalus. Gross total resection along with intraoperative cisternostomy gives excellent results in patients with CPA SOL excision and saves patient from subsequent postoperative CSF diversion.

### Clinical recommendations:

Intraoperative cisternostomy in CPA SOL excision serves a critical role in safe resection of CPA SOL preventing postoperative hydrocephalus and subsequent CSF diversion procedures. Clinicians should utilize this technique for better management of patients with CPA lesions.

### Author`s Contribution:

**TIK:** Supervision, concept and design of the work, critical review of manuscript.

**AG and MNH:** Data interpretation, critically reviewed manuscript.

**HMQ and HT :** Data acquisition data analysis, drafted the manuscript.

All authors agree to Final approval of the version to be published. Agreement to be accountable for all aspects of the work in ensuring that questions related to the accuracy or integrity of any part of the work are appropriately investigated and resolved.
